# Supplementary material for: Venomix: a simple bioinformatic pipeline for identifying and characterizing toxin gene candidates from transcriptomic data
Source: PeerJ. 2018 Jul 31;6:e5361. doi: 10.7717/peerj.5361 (PMC6074769; doi:10.7717/peerj.5361)
Supplement: Supplemental Information 3 [file peerj-06-5361-s003.gz › FinalOutput_E-20/Disintegrin_bitistatin_6/finaltree.pdf]

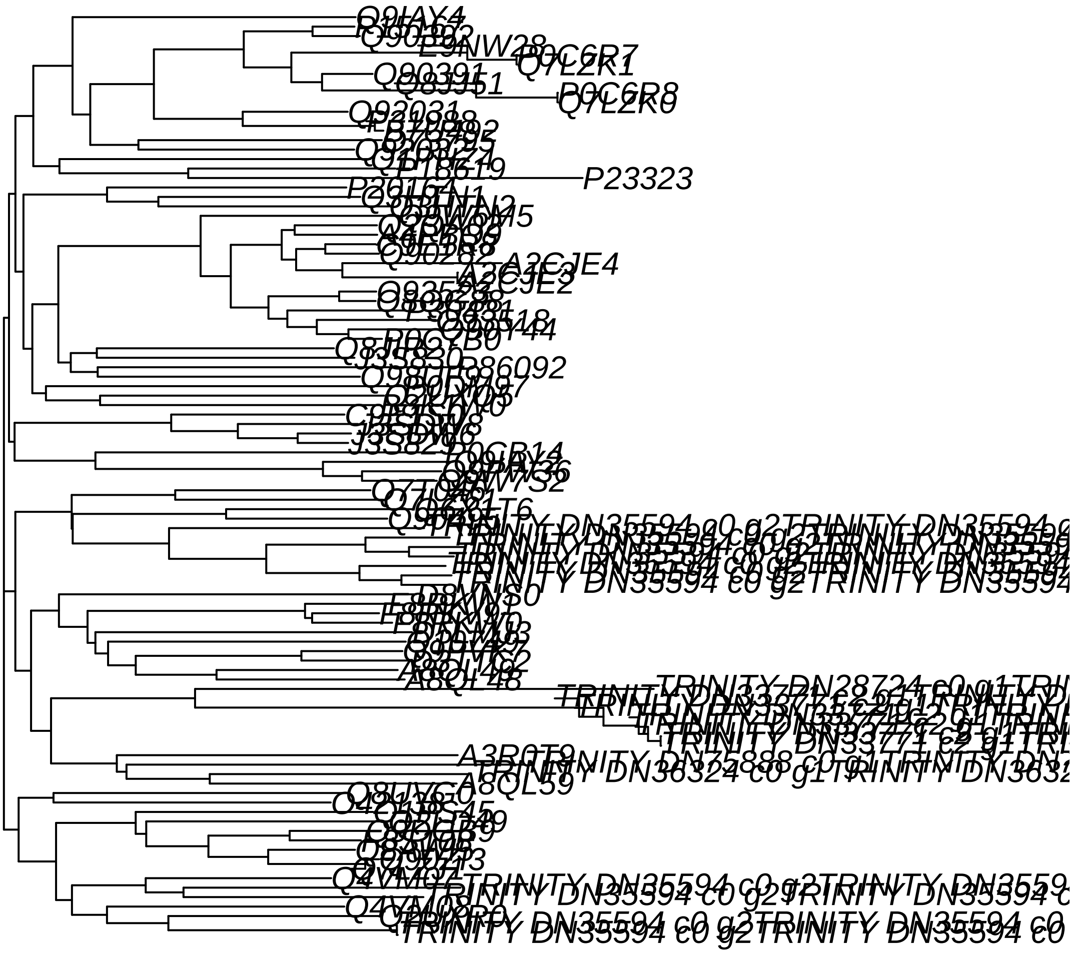

Q9IAY4
E9NW28
P0C6R7
Q7L2K1
Q90391
P0C6R8
Q7L2K0
P23323
A2CJE4
A2CJE2
P86092
D8WIS0
A8QL48
A3R0T9
A8QL59
